# Strategies to preventing pressure injuries among intensive care unit patients mechanically ventilated in prone position: a systematic review and a Delphi study

**DOI:** 10.3389/fmed.2023.1131270

**Published:** 2023-08-14

**Authors:** Zonghua Wang, Jiangshan Fan, Ling Chen, Langlang Xie, Lingfang Huang, Yang Ruan, Xia Xu, Zeping Liang

**Affiliations:** ^1^Department of Clinical Nursing, School of Nursing, Army Medical University, Chongqing, China; ^2^Department of Emergency, The 958th Hospital of PLA, The Affiliated Hospital of Southwest Hospital, Army Medical University, Chongqing, China; ^3^Department of Outpatient, The 79th Hospital of Group Army, Liaoning, China; ^4^Department of Health Management and Geriatric Nursing, Daping Hospital, Chongqing, China; ^5^Department of Nursing, Daping Hospital, Chongqing, China

**Keywords:** intensive care unit, prone position, pressure injuries, strategies, systematic review, Delphi

## Abstract

**Background:**

Although the incidence of pressure injury in the prone position is high for the mechanically ventilated patients in the intensive care unit, evidence-based strategies are still lacking.

**Propose:**

To conduct a systematic review of current evidence, and to propose a series of strategies to prevent pressure injuries among mechanically ventilated patients with prone position in the intensive care unit.

**Methods:**

The study was guided by the Medical Research Council framework. After a systematic review of current evidence of original articles, guidelines, expert consensus and theories, a strategy draft was developed. Then we invited 20 experts to modify and refine these strategies through two rounds of Delphi consensus method.

**Results:**

After two rounds of Delphi process, the importance of coefficient of variation (Cv) and Kendall’s coefficient of concordance in the strategies repository were 0.067 and 0.311, respectively. And the operability of Cv and Kendall’s coefficient of concordance in the strategy draft was 0.055 and 0.294, respectively. Ultimately, we established 31 strategies for including 7 themes (assess risk factors, assess skin and tissue, body position management, skin care, nutrition, preventing medical device-related pressure injuries, education and supervision). In addition, we also developed a strategy framework to clarify our strategies.

**Conclusion:**

According to the Medical Research Council framework, we developed 7 themes and 31 strategies to prevention prone-position pressure injuries among the intensive care unit mechanically ventilated patients. This study was considered to improve the clinical management of pressure injuries among prone position patients in the intensive care unit settings.

## 1. Background

The prone position was widely used in the intensive care unit (ICU) as a lung protective ventilation strategy and has achieved positive clinical outcomes (1, 2). Specifically, ICU patients using the prone position could enhance their oxygenation, reduce the incidence of complications and mortality rates. A recent study found that oxygenation was better in severe COVID-19 patients using the prone position compared with the supine position, and their cumulative adjusted mean difference of SpO_2_/FiO_2_ (409, 95% CI 86–733) and ROX index (26, 95% CI 9–43) were increased during the first 7 days (3). Furthermore, in a multicenter prospective randomized controlled trial including 466 patients with severe ARDS showed that the 28 and 90 days mortality in the prone position was 16.0 and 23.6% respectively, lower than those of 32.8 and 41.0% in the supine position (*p* < 0.001) (4).

However, there are varies of complications in the process of prone ventilation, with pressure injury being the most common (4, 5). A recent scoping review of 27 publications by Julie Sandhu found that the incidence of pressure injuries due to prone position reached 28.5% in 4820 patients in the ICU (6). Additionally, in a study involving 170 mechanically ventilated patients with prone position in ICU, 23 patients developed pressure injuries after prone position (14%). The physical positions of pressure injuries were: chin, 5% (*n* = 8); cheekbones, 6% (*n* = 11), chest, 2% (*n* = 3); trochanter, 1% (*n* = 1) (7).

Pressure injury is defined as local skin and subcutaneous soft tissue injury due to shear or friction alone or in combination, usually occurring at the site of bone protrusion in patients immobilized for an extended period (8). The pathogenesis of pressure injuries is generally recognized to result from tissue ischemia, ischemia–reperfusion injury, and skin injury caused by mechanical load (9, 10). The prevalence of pressure injuries among patients in the wards ranged from 4.7 to 32.1%, with a higher incidence of pressure injuries in ICU (11). In a systematic review (12), the incidence and prevalence of pressure injuries among adult patients in ICU were 10.0–25.9% and 16.9–23.8%, respectively. The cost of treating pressure injuries imposed a huge financial burden on the healthcare system. The annual cost of management pressure injury in the US was approximately $26.8 billion (13), £530 million in the UK, and 1.8 billion dollars in Australia (14). In addition, pressure injury was significantly associated with length of stay, as well as 1.5–2 times greater risk of 30-, 60-, and 90-day readmission compared to patients without pressure injuries (15). Therefore, it is necessary to implement appropriate interventions to prevent pressure injuries and reduce patient distress as well as the financial burden on the healthcare system (16).

Although there has been a lot of articles, expert consensus and guidelines reporting interventions to prevent pressure injuries (17–19), several barriers hinder the success of traditional approaches in clinical practice. First, most of the interventions in the studies focused on pressure injuries in supine patients, and limited data were available on prevention interventions that reduce the incidence of pressure injuries among patients ventilated in prone position (20–22). There might be differences in the strategies of patients adopt supine and prone positions to prevent pressure injuries. For example, the face, thorax, breast region, knees, toes, penis, clavicles, iliac crest, tibial plateau and symphysis pubis were described in the literature as common risk areas for pressure injuries development when patients are in prone position, which were different from sites in supine (23). Secondly, previous studies of patients ventilated in the prone position were mainly concentrated on the wards rather than ICU (24). It was worth noting that patients in the ICU were higher risk of pressure injuries than wards due to severe disease, immobility, poor tissue perfusion and unstable hemodynamics (25). Finally, varies of current preventive interventions for pressure injuries in mechanically ventilated patients with prone position were developed, but not based on evidence and theories (26–28). Without robust to support evidence from previous studies, this limited the development of scientific standards.

To overcome these barriers, this study formulated several strategies based on the Medical Research Council (MRC) framework. The advantage of this framework was used to help researchers work with stakeholders (physicians, nurses, and patients) to identify the core components of complex interventions, and to conduct research from different perspectives and appropriate approaches (22). Currently, the MRC framework has been widely used in health care, as well as other social and economic areas that affect people’s health (29, 30). Therefore, the aim of this study was to identify evidence-based strategies guided by the MRC framework to prevent pressure injuries in prone position patients with mechanically ventilated in ICU and to provide theoretical basis for the future application in clinical nursing work.

## 2. Materials and methods

**Figure 1:**
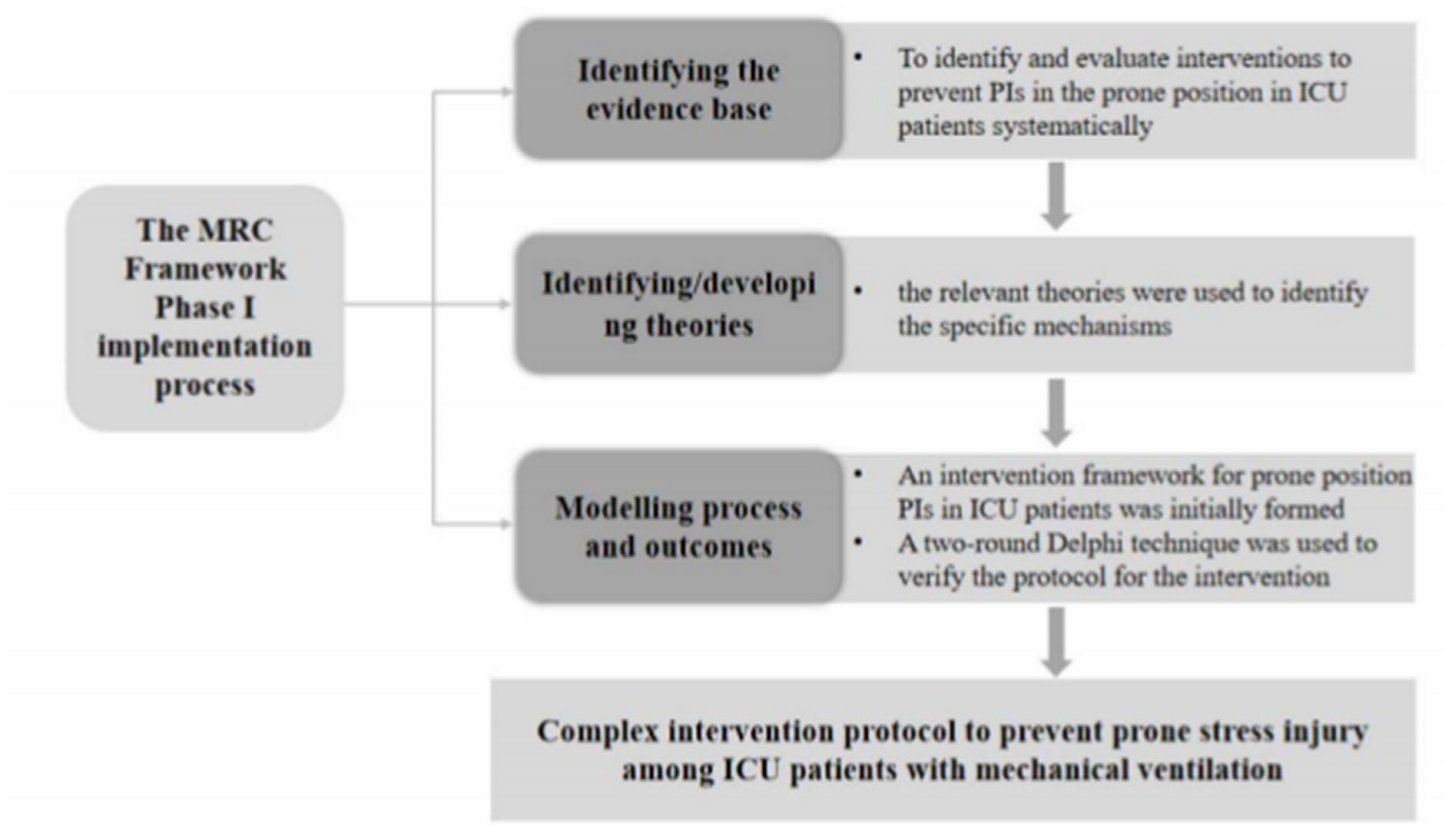
Flow chart of the first phase of the MRC framework.

This study was conducted under the first phase of the MRC framework. First, we performed a systematic review to identify the best evidence. Second, the relevant theories were used to identify the specific mechanisms that could lead to effective interventions, and formed a draft of strategies according to the best evidence summary. Third, we used Delphi technique consisting of two rounds to analyze the strategy draft from the importance and operability, and finally a series of strategies were revealed. A flow chart of the specific methodology was shown in Figure 1.

### 2.1. Step one: identifying the best evidence and theories

#### 2.1.1. Search strategy

According to the Evidence Pyramid “6S” model guided the systematic retrieval of literature (31), using a combination of subject terms and free words. The databases searched included: UpToDate, British Medical Journal Best Practice (BMJ Best Practice), Registered Nurses’ Association of Ontario (RNAO), UK National Institute for Health and Care Excellence (NICE), Guidelines International Network (GIN), National Guideline Clearinghouse (NGC), Scottish Intercollegiate Guidelines Network (SIGN), European Pressure Ulcer Advisory Panel (EPUAP), European Respiratory Society (ERS), Intensive Care Society (ICS), Wound, Ostomy, and Continence Nurses Society (WOCN), Cochrane Library, Joanna Briggs Institute Library, PubMed, Embase, CINAHL, Medlive, CNKI, Wanfang, and China Biomedical Literature Database, etc. Keywords including “prone position ventilation/mechanical ventilation in the prone position/acute respiratory distress syndrome/ARDS,” “pressure ulcer/bedsore/bedsores/pressure sore/pressure sores/bed sores/bed sore/decubitus ulcer/decubitus ulcers/pressure injury/skin breakdown/skin compromise/skin damage,” and “intensive care units/ICU.” Gray literature was also searched in databases including Bielefeld Academic Search Engine (BASE) and Opengrey. The retrieval time was from the establishment of the database to July 30, 2022. We identified existing guidelines, expert consensus, systematic reviews, clinical trials and original studies on the intervention strategies to prevent prone position pressure injuries in mechanically ventilated ICU patients.

#### 2.1.2. Synthesis and grading of evidence

The inclusion criteria of evidence resources were: (a) conducted in adults over 18 years of age who were ventilated in the prone position; (b) evidence content that focused on clinical nursing measures to prevent pressure injury; (c) published in Chinese or English. The exclusion criteria were: (a) studies including children; (b) articles with unavailable full texts; (c) literature with low-quality evaluation results. We followed the update of the clinical guidelines research and evaluation system (AGREE II) to comprehensively judge the recommended levels of the guidelines (32). In addition, the JBI quality evaluation tool was used to evaluate the quality of the systematic evaluation, best practices, and evidence summary. For evidence derived from JBI, the results of its evidence rating and recommendation level were directly applied. Moreover, for the evidence that did not come from JBI, the JBI evidence pre-classification and evidence recommendation level system (2014 version) was adopted (33). The selection of relevant studies was carried out independently by two researchers who had systematic evidence-based training and learning, and when consensus could not be reached, third-party experts in the field were invited to participate in discussions, consultations, and adjudication.

#### 2.1.3. Identifying and developing theories

Within the MRC framework, relevant theories were used to explain the key elements and mechanisms of intervention strategies and how these elements interacted with each other. Identifying relevant theories could facilitate a common understanding of intervention strategies among different stakeholders and identify some key issues where uncertainty exists (22, 34).

### 2.2. Step two: developing the strategy draft

We developed the draft of the strategy based on the feasibility, appropriateness, significance, and effectiveness (FAME) of the intervention for pressure injuries among patients in prone position with mechanical ventilation. The FAME Model was developed by the JBI for Evidence Based Healthcare to complement their inclusive approach to the categorization, synthesis and implementation of evidence (35).

In this context, feasibility referred to evidence that revealed whether it was possible to implement an strategy within a given context (e.g., ICU nurse offered strategies to mechanically ventilated patients in the prone position to prevent pressure injuries) (36). Appropriateness deemed to evidence that demonstrated whether the strategy was ethically or culturally appropriate. In this study, we assessed whether the identified strategies were specifically designed for patients with prone position pressure injuries on mechanical ventilation. Meaningfulness was regarded as evidence that took the form of health care personnel views and experiences of strategies, including their content and ways of implementation. Effectiveness was about assessing the impact of strategies on specific outcomes, such as the incidence of pressure injuries.

### 2.3. Step three: content validation of the evidence-based strategies

#### 2.3.1. Selection of experts

The Delphi survey method was used to select important and operable draft strategies. The selection of experts was based on the following criteria: (a) doctors/nurses from different related fields such as clinical medicine, critical care and nursing; (b) having at least a bachelor’s degree, intermediate or above professional title; (c) at least 5 years of work experience and above; (d) available to participate in two rounds of consulting. According to previous studies, there might be some differences in the sample of participating experts using the Delphi method, but usually included at least 15–30 participants from the same discipline, or 5–10 participants from different professional groups to ensure the scientific and effective results. Therefore, we invited 20 doctors/nurses from different related fields, such as clinical medicine, critical care and nursing to participate in the Delphi process (37).

The draft of the strategies was distributed and returned by email, and the experts were asked to score each strategy. A five-point Likert scale was used to measure the importance and operability of each nursing measure, with five points signifying “very important” or “very strong operability” and one point indicating “very unimportant” or “very weak operability,” respectively, and an expert modification opinions were also set in the evaluation form.

#### 2.3.2. Data analysis

Delphi surveys were sent to experts in two rounds of email between July 2022 and September 2022. In the studies using Delphi technique, consensus is defined as greater than 70% agreement on all components (38, 39). Therefore, we defined consensus as the mean score of importance and operability >4.00 and coefficient of variation (Cv) <0.25. This strategy would be retained, if it score reached the standard. If the score of strategy did not meet the criteria, the retention of the strategy would be determined by expert opinion or panel discussion. For example, based on a systematic review of the literature, our evidence recommended that doctors, nurses, dietitians, and registered respiratory therapists constitute the intervention teams, but the experts seemed to disagree with this approach. Registered respiratory therapists main tasks involve diagnostic and therapeutic interventions for patients with cardiopulmonary diseases under the guidance and supervision of physicians (40). They are more common in foreign hospitals and have been identified to play an important role in the ICU (41, 42). Unfortunately, most hospitals in China did not have professional respiratory therapists, so the experts suggested modifying the content to make the strategies more consistent with the actual clinical practice. After revising and supplementing the strategies draft, we developed a second round of draft and distributed to the experts again.

Data were analyzed using the Excel (43) and version SPSS 26.0 (44) software. The authority coefficient (Cr) of the experts was expressed as the average value of the judgment coefficient and familiarity of the items of the experts, with Cr > 0.80 indicating a high degree of authority (37). The degree of concentration of expert opinions was evaluated using the mean and standard deviation, while the degree of coordination of expert opinions was expressed as the Cv and Kendall’s coefficient of concordance. The Cv <0.25 indicated acceptable expert entry-level differences, and the Kendall’s coefficient of concordance ranged from 0 to 1 (satisfying *p* < 0.05), indicating that the data are statistically significant.

### 2.4. Ethical considerations

The study obtained approval of the Ethics committee of the Chinese People’s Liberation Army Characteristic Medical Center (number: 2020SC30). The patients/participants (legal guardian/next of kin) provided written informed consent to participate in this study.

## 3. Results

### 3.1. Best evidence

**Figure 2:**
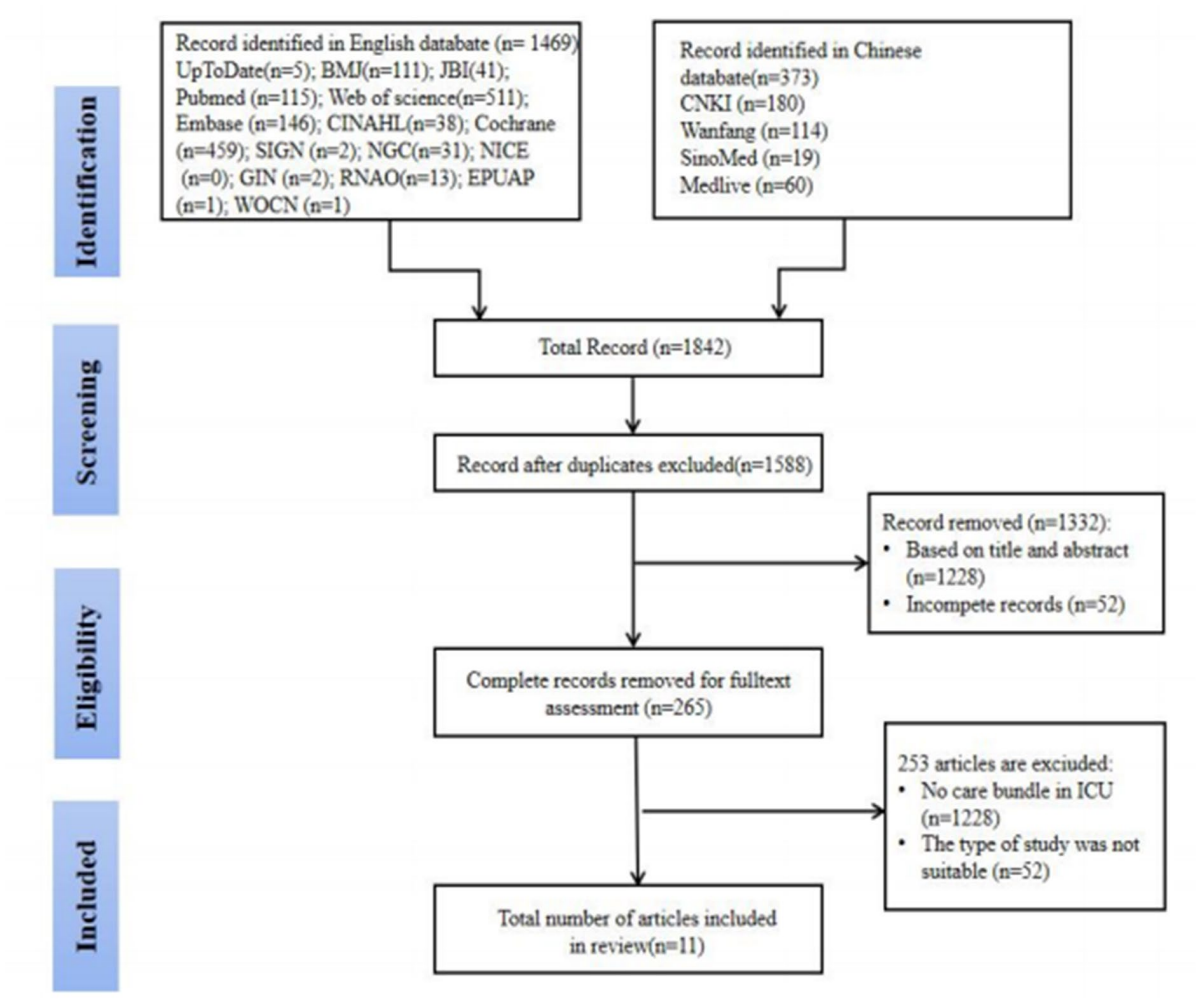
Flow chart of the literature selection.

After the preliminary search, a total of 1,842 articles were obtained, and 11 articles were finally included, including one evidence summary (45), four guidelines (46–49), one expert consensus (50), three systematic evaluations (4, 25, 51), and two cohort studies (52, 53). The literature screening process was shown in Figure 2, and the general characteristics of the included literature were shown in Table 1.

**Table 1:** General characteristics of the included literature.

Author	Year of publication	Study type	Main content	Source of literature
Malhotra (45)	2022	Evidence summary	Prone ventilation for adult patients with acute respiratory distress syndrome	UpToDate
Hashimoto et al. (46)	2017	Guideline	The clinical practice guideline for the management of ARDS in Japan	Medlive
Intensive Care Society (47)	2019	Guideline	Guidance for: Prone Positioning in Adult Critical Care (2019)	Medlive
Griffiths et al. (48)	2019	Guideline	Guidelines on the management of acute respiratory distress syndrome	BMJ Best Evidence
EPUAP (49)	2019	Guideline	Prevention and treatment of pressure ulcers/injuries: clinical practice guideline	Medlive
Wang et al. (50)	2020	Expert consensus	Holistic care for patients with severe coronavirus disease 2019: An expert consensus	CNKI
Patton et al. (51)	2021	Systematic reviews	The effect of prone positioning on pressure injury incidence in adult intensive care unit patients: A meta-review of systematic reviews	PubMed
Bloomfield et al. (4)	2015	Systematic reviews	Prone position for acute respiratory failure in adults	Cochrane Library
Moore et al. (25)	2020	Systematic reviews	Prevention of pressure ulcers among individuals cared for in the prone position: lessons for the COVID-19 emergency	PubMed
Alderden et al. (52)	2022	Cohort study	Pressure Injury Risk Assessment and Prevention in Patients With COVID-19 in the Intensive Care Unit	PubMed
Chao et al. (53)	2021	Cohort study	Study on the design and application of prone positioning ventilation treatment checklist inpatients with severe ARDS	CNKI

Four guidelines were included in this study. One was from BMJ Best Evidence (48), and the other three were from Medlive (46, 47, 49). Two evaluators independently evaluated all the guidelines, with high consistency among the evaluators. The percentage of standardization in all fields and the overall quality evaluation scores of the guidelines were shown in Supplementary Table S1.

One expert consensus from CNKI was included in this study, which indicated that the views were derived from influential experts in this field (50). In addition, the views proposed in this article were centered on the interests of the relevant population, logical, and consistent with other literature. Its conclusions were based on the results of the analysis and existing literature. The overall quality of the literature was relatively high, so we considered inclusion in this study. Furthermore, three systematic reviews were included: two from PubMed (25, 51) and one from the Cochrane Library (4). The items in these four papers were all evaluated as “yes,” indicating high research quality. The specific evaluation process was shown in Supplementary Table S2. We included an RCT from an evidence summary (54). All the entries in the study were evaluated as “yes,” the study design was relatively complete, and the overall quality was high. Finally, three cohort studies were included: one from CNKI (53), one from PubMed (52), and the other from an evidence synthesis (55). The overall design of these studies was relatively complete, and the overall quality was relatively high. The specific evaluation processes were shown in Supplementary Table S3.

Eventually we formed 32 best evidence including 7 themes (assess risk factors, assess skin and tissue, body position management, skin care, nutrition, preventing medical device-related pressure injuries, education and supervision).

### 3.2. Theory of preventing prone pressure injuries in mechanically ventilated ICU patients

In this study, the predictive models constructed by Tschannen (56) and Coleman (57) were used to explain the mechanism of pressure injuries with prone position in mechanically ventilated patients in ICU due to they were considered the most appropriate models, and provided guidance for the draft of the strategy.

Pressure, skin status and poor perfusion were the direct reasons of pressure injuries in patients according to the conceptual model of Tschannen and Coleman (56, 57). Permanently bedridden patients were increased risk of pressure injury, which have negatively affects to skin and tissue tolerance due to constant pressure. Patient’s skin would appear inflammation and ulceration if the pressure was not relieved. It also caused muscle necrosis when the wound invasions to the deep tissue. In addition, malnutrition increased the risk of pressure injuries. Malnutrition was associated with defective blood perfusion, mainly due to poor oxygenation, limited perfusion, and high water content.

In addition, Tschannen’s prediction model of pressure injuries emphasized that indirect factors including demographics characteristics of patients, environmental context factors and factors directly related to the hospital episode of care also affect the incidence of pressure injuries except to direct factors (56). Although limited data were available, demographic characteristics such as age, gender, marital status, and level of consciousness could predict pressure injuries. Environmental context factors include type of unit, knowledge and skill level of nurses, nursing workload. For instance, the type of unit patients were admitted to was associated to pressure injuries, with ICU or surgical hospitalization being at greater risk for pressure injuries. Furthermore, factors directly related to hospital episode care, including patient admission diagnosis, severity of illness, length of stay, were estimated for pressure injuries.

There are few intervention strategies including environmental context factors and factors directly related to hospital-care events for pressure injuries in mechanically ventilated ICU patients in prone position currently. The discovery of this is not surprising, it is due to the complexity and dynamics of pressure injuries. In practice, the identification of appropriate intervention strategies should consider practical concerns. Therefore, combined with the results of the best evidence summary and theoretical review, we formulated the strategy draft including 7 themes (assess risk factors, assess skin and tissue, body position management, skin care, nutrition, preventing medical device-related pressure injuries, education and supervision) and 31 strategies for pressure injuries in mechanical ventilation patients with prone position in ICU.

### 3.3. Content validation of the strategies

#### 3.3.1. General information of experts

In the study, the average age of experts was 41.70 ± 7.07 years, and their average working years in their professional fields was 20.85 ± 8.45 years. Most of the experts had at least a bachelor’s degree (Table 2). The proportion of the questionnaires recovered in the two rounds was 100%, indicating the high enthusiasm of the experts to participate in this study. In general, Cr was 0.7 meaning that the experts considered the results to be more reliable. Moreover, Cr during the two rounds of consultation was 0.91, which showed good reliability of our research results.

**Table 2:** General information of experts of the experts (*n* = 15).

Items	Classification	Number	Proportion (%)
Age	<40 years	3	20.0
	40–50 years	6	40.0
	>50 years	6	40.0
Gender	Male	1	6.7
	Female	14	93.3
Education	Master	10	66.7
	Doctor	5	33.3
Professional position	Intermediate certificate	4	26.7
	Deputy senior position	6	40.0
	Senior position	5	33.3
Years of working	<20 years	4	26.7
	20–30 years	9	60.0
	>30 years	2	13.3
Field of research	Medical education	2	13.3
	Nursing education	6	40.0
	Nursing management	3	20.0
	Pedagogy	4	26.7

After two rounds of consultation in this study, the Cv of importance were 0.144 and 0.067, and the Cv of operability were 0.137 and 0.055. Moreover, the Kendall’s coefficient of concordance for the importance and operability of the items was 0.165 and 0.311 and 0.162 and 0.294, respectively. All the above data showed that the expert opinions are well coordinated. The relevant data of each round and each level of strategies were shown in Table 3.

**Table 3:** The degree of coordination of expert opinions in strategies for preventing pressure injuries among ICU patients mechanically ventilated in prone position.

Rounds	Level	Importance		Operability	
		Cv	Kendall’s W	Cv	Kendall’s W
First round	First-level item	0.150	0.178	0.155	0.147
	Second-level item	0.143	0.184	0.133	0.172
	Total	0.144	0.165	0.137	0.162
Second round	First-level item	0.044	0.210	0.044	0.262
	Second-level item	0.072	0.334	0.058	0.300
	Total	0.067	0.311	0.055	0.294

#### 3.3.2. The first round of expert questionnaire consultation

After the first round of expert questionnaires were collected, some items were modified based on experts’ opinions and discussion of research team members. In total, we modified the content of nine strategies and added and supplemented the content of five strategies. For example, experts suggested that “2.2 Assessors: the nurse on duty should evaluate the skin and organization, and record the documents” to “2.2 Assessors: The nurse on duty will assess the condition of the skin and tissue, record the paperwork, and the head nurse or group nurse will judge the skin condition and writing quality.” The purpose of this strategy was mainly from the leadership level to strengthen the supervision and management. Moreover, experts recommend changing the “7.2 Quality improvement project” to “7.2 Quality control index.” Experts believed that patients with mechanical ventilation should achieve the treatment goal as the primary goal, and the skin care goal should be subordinate to the treatment goal. Thus, the incidence of pressure injury with prone position could be used as a quality index rather than a quality improvement project. In addition, we also adjusted the order of some strategies. For instance, several experts suggested that “3.1 Keep the respiratory tract unobstructed; 3.2 Time for body position replacement; 3.3 Group participation in repositioning.” should be adjusted to “3.1 Time for body position replacement; 3.2 Group participation in repositioning; 3.3 Keep the respiratory tract unobstructed.” Experts deemed that the adjusted order was more in line with the implementation logic of clinical care, and we decided to adopt the opinion after an in-depth discussion.

#### 3.3.3. The second round of expert consultation

We found that the experts’ responses were generally consistent after the second round, and only a few parts of the items were modified. After considering the expert opinions and suggestions as well as research group discussions, 7 themes and 31 strategies were developed to prevent pressure injuries in mechanically ventilated patients with prone position in ICU (Supplementary Table S4).

**Figure 3:**
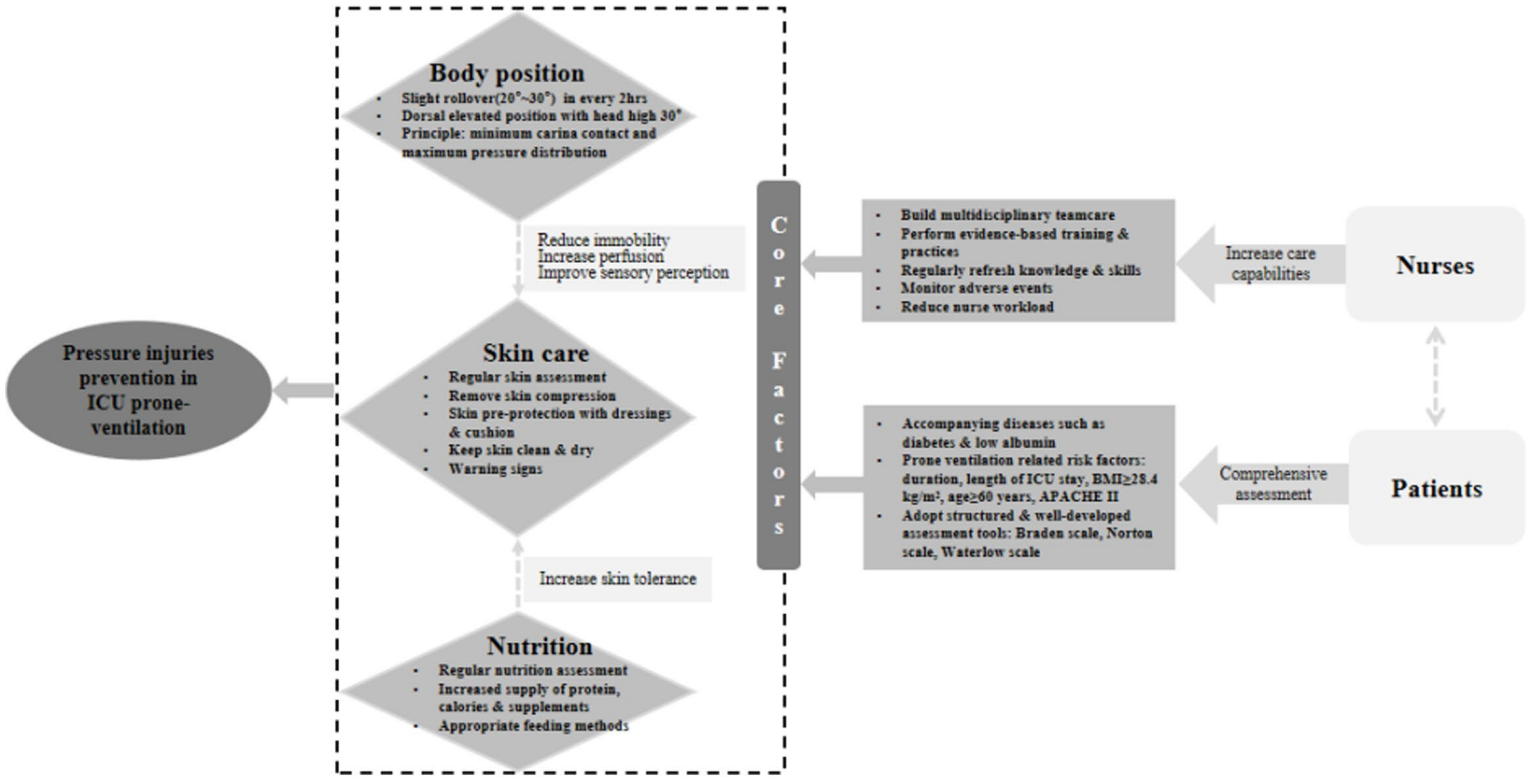
Strategy framework for pressure injuries among mechanically ventilated patients in ICU.

After completing the two rounds of Delphi process, we organized in-depth group discussion again according to the results and clinical practice. To make these easier for medical staff, we integrated 31 strategies into a strategy framework as shown in Figure 3. The boxes in the dashed line describe the core topics of pressure injuries among mechanically ventilated patients with prone position in ICU and the effects on pressure injuries. Among them, the prevention of pressure injuries was better when the three core topics in this study were well-controlled. Meanwhile, two of the core topics (body position and nutrition) have, respectively, connected the third core topics (skin care) with dashed lines, indicating that both will indirectly affect skin care. Moreover, the education and supervision of nurses and the general demographic characteristics of patients are also necessary as they influence each other.

## 4. Discussion

The detailed process for the formulation of theory-based and evidence-based strategies that aimed to the mechanical ventilated patients with prone position in ICU is reported in this article. Following the MRC framework, strategies in this study connect existing evidence-based content from high-quality researches, and standpoints from healthcare professionals. There are 31 strategies to prevent pressure injuries to mechanical ventilated patient with prone position in the ICU. These strategies relate to 7 main themes: assess risk factors, assess skin and tissue, body position management, skin care, nutrition, preventing medical device-related pressure injuries, education and supervision. Figure 3 captures the relationship between these topics, and provides a strategy framework to pressure injuries among mechanical ventilated patients with prone position in the ICU.

In this strategy framework, body position, skin care and nutrition were regard as the core factors. Mechanically ventilated patients in the ICU have severe illness and long hospital stays. The friction between skin and bed sheet is increased due to bedridden for a long time. This pressure is not relieved for a long time, which is easy to make patients suffer from pressure injuries (58). Body position strategies such as reposition the patient every 2 h, and raising the head of the bed could overcome this problem (59).

Skin care is defined as assessing the condition, integrity, temperature, humidity of the patient’s skin, and removing dirt, sebum, and other substances from the skin immediately (60). When the patient is prone, it is important to assess the skin as soon as possible. This is regarded as part of each risk assessment, according to the patient’s level of risk of pressure injuries, during the nurse’s shift and before the end of nursing work (25). Skin assessment was performed in clinical practice using the “Braden” scale, the “Norton” scale, the “Waterlow” pressure injuries risk assessment scale, or the ICU-specific assessment scale (Cubbin and Jackson scale). However, there was a lack of skin assessment tools for pressure injury among mechanically ventilated patients with prone position (61, 62). In addition, We have discussed the main recommendations for pressure injuries prevention in ICU Mechanically ventilated patients in prone position from the latest version of Prevention and Treatment of Pressure Ulcers/Injuries: Clinical Practice Guideline, International Version (23), including the preparation phase, prone position care, and supine repositioning care. These recommendations emphasize the need for skin care. For example, skin assessment is recommended before placing the patient in the prone position. When the patient is in the prone position, it is recommended to keep the skin clean.

Nutrition plays an important role in the prevention and treatment of pressure injuries. Malnutrition and weight loss have been linked to pressure injuries (63, 64). An Australian study conducted in ICU and wards found that, for adults with malnutrition, the odds ratio of developing a pressure injuries was 2.6 (95% CI, 1.8–3.5) in ICU, and 2.0 (95% CI, 1.5–2.7) in wards (65). In addition, overweight or obese patients often further increase the risk of stress injuries due to reduced mobility. The microorganisms attracted by the moist environment generated by skin folds could lead to infection and tissue damage (66). European Pressure Ulcer Advisory Panel (EPUAP) recommends a comprehensive nutritional assessment of adults at risk for pressure injuries and malnutrition (23). A study conducted in Florida examined the use of a nutritional regimen in 100 patients age 60 or older with stage II and/or stage III pressure injuries. The study concluded that having a nutritional assessment was positively associated with pressure injuries cure rates (67).

Nurse factors included education and training, multidisciplinary team care, and reduction of nurse workload appropriately. However, it was worth that in Dana Tschannen’s study (56), higher nursing workload was thought to be associated with lower pressure injuries in patients. This was inconsistent with the results of our study and might be due to the different hospitals in different countries. In addition, the prevention of medical device injury is included in the topic of nurse factors, because we thought that nurses would care for patients wearing ventilators and other medical devices or tubes every day, and it is necessary to master methods to reduce the risk of pressure injuries caused by medical devices to patients. For example, by changing the position of the device to minimize pressure and shear force, and by using preventative dressing (60, 68). Patient factors are mainly reflected in the integrated risk assessment of patient demographic characteristics. The risk assessment result denotes what level of risk an individual has for pressure injuries development and serves as a trigger for nurses to initiate prevention strategies to deter pressure injuries occurrence (69).

The study has three advantages. First, it is to follow the MRC framework and use a systematic review of the literature and theories approach to develop evidence-based strategies. In addition, experts in the fields of clinical medicine, critical care and nursing rated the importance and operability of strategies. Previous studies have confirmed that expert panels provide valid representations of the views of the fields they represent. Therefore, specialists and clinical nurses are involved in the confirmation process of the effectiveness of strategies, ensuring that they are clinically feasible and more acceptable to clinical nurses. Finally, this research summarized strategies into a framework that could be help healthcare professionals to understand these strategies.

Nevertheless, there were some limitations to this study. First, the use of Delphi technology could be a limitation. Experts provide a wide range of opinions based on their knowledge and clinical experience, which could be subjective and bias the results, and we will use more objective data to revise and refine the findings. Secondly, we invited experts from China, and our findings might be more applicable to the medical environment in China due to regional and cultural differences. Finally, we only completed the first step of the MRC framework, and further clinical practices are needed to validate these strategies in the future.

## 5. Conclusion

The findings of this study revealed a range of strategies to prevention pressure injuries in mechanical ventilation patients with prone position in ICU which based on the MRC framework, and thus improved clinical outcomes for the patients.

## Data availability statement

The original contributions presented in the study are included in the article/Supplementary material, further inquiries can be directed to the corresponding authors.

## Ethics statement

The study obtained approval of the Ethics committee of the Chinese People’s Liberation Army Characteristic Medical Center (number: 2020SC30). The patients/participants (legal guardian/next of kin) provided written informed consent to participate in this study.

## Author contributions

ZW, LC, and XX conceived the study. ZW, LC, LX, and JF completed the whole experiments. JF, ZW, and LH carried out the literature review and screening. LC, LH, and YR performed the data extraction. ZW and JF drafted the manuscript. XX and ZL revised the manuscript. All authors have read and approved the final version of the manuscript.

## Funding

This work was supported by the Chongqing Technology Innovation and Application Development Special Programme (Grant No. cstc2019jscx-msxm-X0286), Chongqing Science and Health Joint Medical Research Programme (Grant No. 2020FYYX139), and The Military Medical Science and Technology youth Cultivation Programme (Grant No. 21QNPY020).

## Conflict of interest

The authors declare that the research was conducted in the absence of any commercial or financial relationships that could be construed as a potential conflict of interest.

## Publisher’s note

All claims expressed in this article are solely those of the authors and do not necessarily represent those of their affiliated organizations, or those of the publisher, the editors and the reviewers. Any product that may be evaluated in this article, or claim that may be made by its manufacturer, is not guaranteed or endorsed by the publisher.
