# Supplementary material for: Strategies to preventing pressure injuries among intensive care unit patients mechanically ventilated in prone position: a systematic review and a Delphi study
Source: Front Med (Lausanne). 2023 Aug 14;10:1131270. doi: 10.3389/fmed.2023.1131270 (PMC10461099; doi:10.3389/fmed.2023.1131270)
Supplement: Supplementary file 1 [file Table_1.DOCX]

Supplementary Material

**Strategies to Preventing pressure injuries among intensive care unit patients mechanically ventilated in prone position: a systematic review and a Delphi study**

**Zonghua Wang^1#^, Jiangshan Fan^1#^, Ling Chen^2^, Langlang Xie^1^, Lingfang Huang^1^, Yang Ruan^3^, Xia Xu^4*^, Zeping Liang^5*^**

*** Correspondence:**For manuscript inquires, contact:

Dean Xia Xu at: [406938690@qq.com](mailto:406938690@qq.com)

Lecturer Zeping Liang at: [419624966@qq.com](mailto:419624966@qq.com)

**^#^** Zonghua Wang and Jiangshan Fan contributed equally to the manuscript and shared first authorship

# Supplementary Tables

**Supplementary Table 1 Results of quality assessment of included guidelines**

| **Included literature** | **Standardized percentage scores across the domains（%）** | | | | | | **Number of projects with a score ≥60%** | **Number of projects with a score ≥30%** | **Level** |
| --- | --- | --- | --- | --- | --- | --- | --- | --- | --- |
|  | **Scope and Purpose** | **participants** | **stringency** | **clarity** | **applicability** | **independence** |  |  |  |
| Griffiths MJD^[46]^ | 81.0 | 85.7 | 80.4 | 66.7 | 71.4 | 85.7 | 6 | 6 | A |
| EPUAP^[47]^ | 95.2 | 71.4 | 80.4 | 85.7 | 85.7 | 92.6 | 6 | 6 | A |
| Hashimoto S^[44]^ | 57.1 | 66.7 | 78.6 | 71.4 | 82.1 | 85.7 | 5 | 6 | B |
| Intensive Care Society^[45]^ | 85.7 | 95.2 | 53.6 | 76.1 | 85.7 | 90.5 | 5 | 6 | B |

**Supplementary Table 2 Results of quality assessment of included systematic reviews**

| **Items** | **Bloomfield R^[4]^** | **Patton D^[49]^** | **Moore Z^[25]^** |
| --- | --- | --- | --- |
| 1.Are the evidence-based questions raised clear and clear? | Yes | Yes | Yes |
| 2.Are the inclusion criteria for the literature appropriate? | Yes | Yes | Yes |
| 3.Is the retrieval strategy appropriate? | Yes | Yes | Yes |
| 4.Is the source of the research paper appropriate? | Yes | Yes | Yes |
| 5.Are the literature quality evaluation criteria used appropriate? | Yes | Yes | Yes |
| 6.Do two or more evaluators independently complete the literature quality evaluation? | Yes | Yes | Yes |
| 7.Are certain measures taken to reduce error when extracting data? | Yes | Yes | Yes |
| 8.Is the integrated / pooled study approach appropriate? | Yes | Yes | Yes |
| 9.Is the possible publication bias assessed? | Yes | Yes | Yes |
| 10.Are there recommendations for policy and (or) practices supported by reported data? | Yes | Yes | Yes |
| 11.Are there appropriate recommendations for specific directions of further future research? | Yes | Yes | Yes |

**Supplementary Table 3 Results of quality assessment of included cohort studies**

| **Items** | **Alderden J^[50]^** | **Reignier^[53]^** | **Gao^[51]^** |
| --- | --- | --- | --- |
| 1.Does each group of study subjects have similar characteristics and come from the same population? | Yes | Yes | Yes |
| 2.Are exposure factors assessed in the same way or randomly assigned exposure to non-exposure? | Yes | Yes | Yes |
| 3.Is the evaluation method of exposure factors effective and credible? | Yes | Yes | Yes |
| 4.Are the confounding factors considered? | No | No | No |
| 5.Are measures taken to control the confounders? | No | No | No |
| 6.Does it describe no outcome at the beginning of the exposure or study? | Yes | Yes | Yes |
| 7.Is the evaluation method of outcome indicators effective and credible? | Yes | Yes | Yes |
| 8.Is the follow-up time long enough to observe the outcome occurrence? | Yes | Yes | Yes |
| 9.Are the reasons for the loss to follow-up described and analyzed? | Yes | Yes | Unclear |
| 10.Are there measures taken to deal with lost visits? | Yes | Yes | Unclear |
| 11.Is the method of data analysis appropriate? | Yes | Yes | Yes |

**Supplementary Table 4 The intervention strategies for preventing pressure injuries among ICU patients mechanically ventilated in prone position**

| **Item of first-level** | **Item of second-level** | **Importance** | | | **Operability** | | |
| --- | --- | --- | --- | --- | --- | --- | --- |
|  |  | **Mean±Standard Deviation** | **Cv** | **Full score ratio** | **Mean±Standard Deviation** | **Cv** | **Full score ratio** |
| **1. Assess risk factors** |  | 4.75±0.444 | 0.094 | 75% | 5 | 0 | 100% |
|  | **1.1 Assess high-risk groups:** age≥60 years, male, BMI > 28.4kg/m^2^, malnutrition, history of pressure injury, history of diabetes/renal failure/ cardiovascular disease, wet/ dry/ edema, use of vasopressor/ sedation/ sedation pain medication. | 5 | 0 | 100% | 4.70±0.470 | 0.100 | 70% |
|  | **1.2 Assessment of risk factors:** duration of prone ventilation, duration of body position/frequency of change, length of ICU stay, gender, age, BMI, Glasgow Coma Scale (GCS), Acute Physiology and Chronic Health Score (APACHE II). | 4.85±0.367 | 0.076 | 85% | 4.65±0.489 | 0.105 | 65% |
|  | **1.3 Assessment tools:** Effective, reliable professional assessment tools are recommended for evaluation using structured methods and combined with clinical judgement to assess outcomes. Using the "Braden" scale, the "Norton" scale, the "Waterlow" pressure ulcer risk assessment scale, or the ICU-specific assessment scale (Cubbin & Jackson scale). No risk assessment tools for pressure injuries in the prone position are currently available | 4.45±0.510 | 0.115 | 45% | 4.70±0.470 | 0.100 | 70% |
| **2. Assess skin and tissue** |  | 5 | 0 | 100% | 4.90±0.308 | 0.063 | 90% |
|  | **2.1 Timing of Assessment:** Perform a comprehensive skin and tissue assessment before and after prone ventilation, or during nurse shifts. | 4.70±0.470 | 0.100 | 70% | 4.50±0.513 | 0.114 | 50% |
|  | **2.2 Assessor:** The nurse on duty will assess the condition of the skin and tissue, record the paperwork, and the head nurse or group nurse will judge the skin condition and writing quality. | 4.45±0.510 | 0.115 | 45% | 5 | 0 | 100% |
|  | **2.3 Assess the skin and tissue status of high-risk sites:** Assess changes in skin and tissue mucosal integrity, temperature, color, humidity at high-risk sites, such as auricle, forehead, face, mandible, sternum, female chest, iliac crest, male perineum, tibia, knee, and toes, and use the National Pressure Ulcer Advisory Panel (NPUAP) to grade the skin at the injury site. | 4.95±0.224 | 0.045 | 95% | 4.95±0.224 | 0.045 | 95% |
| **3. Body position management** |  | 5 | 0 | 100% | 5 | 0 | 100% |
|  | **3.1 Time for body position replacement:** Determining the position change time according to the patient's treatment goals, disease status, skin and tissue tolerance, comfort, al. | 4.75±0.444 | 0.094 | 75% | 4.95±0.224 | 0.045 | 95% |
|  | **3.2 Group participation in repositioning:** At least 5 doctors and nurses are required to participate in each position change. One doctor or nurse stands at the head of the bed, and two medical staff on each side of the bed are responsible for turning over and protecting the pipeline on the same side. The medical staff at the head of the bed is also responsible for overall coordination. | 4.40±0.503 | 0.114 | 40% | 4.55±0.510 | 0.112 | 55% |
|  | **3.3 Keep the respiratory tract unobstructed:** Before changing the position, clean the secretions in the oral cavity, nasal cavity and airway, and use high flow or 100% oxygen to oxygenate the patient; maintain the artificial airway balloon pressure of 25-30cmH_2_O. | 4.80±0.410 | 0.085 | 80% | 4.50±0.513 | 0.114 | 50% |
|  | **3.4 The way that the body bits are transformed:** Use the “silkworm baby style” (the method of wrapping the patient with bed sheets) to turn over to change the body position. | 4.90±0.308 | 0.063 | 90% | 5 | 0 | 100% |
|  | **3.5 The principle of body position placement:** Body positioning follows the principles of minimal bony prominence contact and maximal pressure distribution. | 5 | 0 | 100% | 5 | 0 | 100% |
|  | **3.6 Frequency of turning over:** After prone position, at least every 2h intermittent slight turning over for decompression (20°-30°). | 4.50±0.513 | 0.114 | 50% | 4.70±0.470 | 0.100 | 70% |
|  | **3.7 Raise the Angle of the bed：**The head is high and the feet are low in the prone position, and the head of the bed is kept at a height of 15-30°. | 5 | 0 | 100% | 5 | 0 | 100% |
|  | **3.8 Duration of the body position：**The duration of the prone position is generally 6-8h/day, and can last for more than 12h-16h/d if the patient can tolerate it, and the time of turning over is recorded on the nursing document. | 4.95±0.224 | 0.045 | 95% | 4.70±0.470 | 0.100 | 70% |
|  | **3.9 Observe vital signs：**Closely observe the patient's condition before and after turning over, evaluate and record vital signs by a specific person in charge, and deal with problems in a timely manner. | 4.95±0.224 | 0.045 | 95% | 5 | 0 | 100% |
| **4. Skin care** |  | 4.95±0.224 | 0.045 | 95% | 5 | 0 | 100% |
|  | **4.1 The compression site use prophylactic dressings:** Observe the pressure on the skin, and use hydrocolloids, transparent film dressings, foam dressings and other decompression dressings or decompression devices to protect key pressure areas. ① Head: Change the head direction every two hours and place cushions under the head to prevent pressure sores; ② apply eye cream to protect the eyeball; ③ provides additional support for adding soft pillows to the chest, shoulders, hips, and legs. | 5 | 0 | 100% | 5 | 0 | 100% |
|  | **4.2 Clean and dry:** Keep skin clean and dry, avoid hot water, alkaline soaps, do not massage or scrub hard on fragile skin, use warm water or pH-balanced non-sensitive skin cleansers to protect skin. | 4.95±0.224 | 0.045 | 95% | 4.80±0.410 | 0.085 | 80% |
|  | **4.3 Sign:** Post prominent pressure injury warning signs by the bedside. | 4.50±0.513 | 0.114 | 50% | 4.90±0.308 | 0.063 | 90% |
|  | **4.4 Replace pollutants:** Change soiled or moist sheets and dressings promptly. | 4.60±0.503 | 0.109 | 60% | 4.65±0.489 | 0.105 | 65% |
| **5. Nutrition** |  | 4.80±0.410 | 0.855 | 80% | 4.90±0.308 | 0.063 | 90% |
|  | **5.1 Nutritional types:** Provide patients with high protein, high vitamin, high calorie diet to increase nutrition. | 5 | 0 | 100% | 4.60±0.503 | 0.109 | 60% |
|  | **5.2 Feeding patterns:** An appropriate feeding route was chosen based on the patient's tolerability. Enteral nutrition is used in patients who cannot be taken orally and parenteral nutrition is provided to patients when their enteral nutrition is intolerance or enteral nutrition cannot meet the criteria. | 4.80±0.410 | 0.855 | 80% | 5 | 0 | 100% |
|  | **5.3 Gastrointestinal tolerance:** When patients adopt gastric tube feeding, it is necessary to pump the gastric contents before the prone position, and monitor the gastric residual amount, while controlling the feeding rate, in addition to the appropriate use of gastric kinetic drugs, so as to improve the feeding tolerance. | 4.65±0.587 | 0.126 | 75% | 4.50±0.513 | 0.114 | 50% |
| **6. Preventing medical device-related pressure injuries** |  | 5 | 0 | 100% | 4.85±0.366 | 0.076 | 85% |
|  | **6.1 Correct fixed:** Select the appropriate medical device for the patient and fix it correctly. Firstly, the catheter was fixed by an elevated platform method. Secondly, placing two fingers between the fixing band of the tracheotomy tube and the skin was recommended. | 4.75±0.444 | 0.094 | 75% | 5 | 0 | 100% |
|  | **6.2 Management of instruments and pipelines:** When the patient changes the body position, the medical device should be managed and fixed by a special person, while ensuring appropriate tightness and avoiding direct compression of the skin tissue. Meanwhile, it was suggested that the drain pipe would need to be clamped and fixing the catheter again before changing the position. | 4.95±0.224 | 0.045 | 95% | 4.95±0.224 | 0.045 | 95% |
|  | **6.3 Check the skin:** Temporary removal of a medical device without affecting patient care to assess the skin under and around it. | 4.85±0.366 | 0.076 | 85% | 5 | 0 | 100% |
|  | **6.4 Use dressing:** Prophylactic use of a decompression dressing. If the dressing is moist, dirty, loose, or damaged, it should be replaced promptly. | 4.45±0.510 | 0.115 | 45% | 5 | 0 | 100% |
| 1. Education and Supervision |  | 0.475±0.444 | 0.094 | 75% | 4.55±0.510 | 0.112 | 55% |
|  | **7.1 Multi-disciplinary cooperation:** Assemble a multidisciplinary team of doctors, nurses and nutritionists. | 5 | 0 | 100% | 4.30±0.470 | 0.109 | 30% |
|  | **7.2 Quality control index:** Medical institutions/departments regard the incidence of pressure injury in prone-position mechanically ventilated patients as a quality control indicator. | 4.45±0.510 | 0.115 | 45% | 4.95±0.224 | 0.045 | 95% |
|  | **7.3 Continuing learning:** The department regularly conducts special lectures and discussions on the knowledge of pressure injury related to prone position ventilation. | 4.35±0.587 | 0.135 | 40% | 4.35±0.489 | 0.112 | 35% |
|  | **7.4 Training and assessment:** Systematic education and training for medical staff, including: risk identification, skin assessment, staging of stress injury, selection of decompression tools / dressings, and how to flip the prone position. Only the medical staff who pass the assessment can participate in the treatment and care of prone position ventilation. | 4.70±0.470 | 0.100 | 70% | 4.95±0.224 | 0.045 | 95% |
|  | **7.5 Psychological care:** Medical staff need to strengthen communication with patients and their families, and provide psychological care to patients. | 4.45±0.510 | 0.115 | 45% | 4.85±0.366 | 0.076 | 85% |

*Full score ratio: represents the proportion of experts choosing a score of 5 points
